# The role of sarcopenia in all-cause mortality prediction among community-dwelling adults with eGFR 15–89 mL/min/1.73 m^2^

**DOI:** 10.1515/med-2026-1531

**Published:** 2026-10-22

**Authors:** Kaili Chang, Bowen Zhu, Xiaoqiang Ding, Weiming Wang, Yan Dai

**Affiliations:** Department of Nephrology, Ruijin Hospital, Shanghai Jiao Tong University School of Medicine, Shanghai, China; Department of Nephrology, Zhongshan Hospital, Fudan University, Shanghai, China; Shanghai Medical Center of Kidney Disease, Shanghai, China; Shanghai Key Laboratory of Kidney and Blood Purification, Shanghai, China; Shanghai Institute of Kidney and Dialysis, Shanghai, China

**Keywords:** CHARLS, sarcopenia, all-cause mortality, NRI, IDI

## Abstract

**Objectives:**

Sarcopenia is common among individuals with impaired kidney function and is associated with poor quality of life and adverse clinical outcomes. This study aimed to investigate the association between sarcopenia and all-cause mortality among Chinese community-dwelling middle-aged and older adults with an estimated glomerular filtration rate (eGFR) of 15–89 mL/min/1.73 m^2^.

**Methods:**

Using data from the China Health and Retirement Longitudinal Study (CHARLS), we included 2,526 participants aged 45–85 years with an eGFR of 15–89 mL/min/1.73 m^2^ who were followed from 2011 to 2018. Because albuminuria and other markers of kidney damage were unavailable, the study population was operationally defined according to eGFR criteria. Cox proportional hazards regression models were used to evaluate the association between sarcopenia and all-cause mortality. Exploratory analyses assessed the incremental prognostic value of sarcopenia using 5-year time-dependent area under the receiver operating characteristic curve (AUC), integrated discrimination improvement (IDI), and continuous net reclassification improvement (NRI).

**Results:**

Among the 2,526 participants included in the final analysis, the prevalence of sarcopenia was 28.1 % (335/1,194) in men and 41.4 % (551/1,332) in women. After adjustment for potential confounders, sarcopenia was associated with a significantly higher risk of all-cause mortality in women (HR, 1.82; 95 % CI, 1.26–2.63). In exploratory analyses, the addition of sarcopenia to the clinical variable-based model modestly improved 5-year mortality risk discrimination in women, increasing the time-dependent AUC from 0.615 (95 % CI, 0.561–0.670) to 0.650 (95 % CI, 0.599–0.700). The IDI was 0.008 (95 % CI, 0.003–0.014; p=0.006), whereas the continuous NRI was 0.236 (95 % CI, −0.055–0.417; p=0.090).

**Conclusions:**

Sarcopenia was associated with an increased risk of all-cause mortality among women with an eGFR of 15–89 mL/min/1.73 m^2^. Sarcopenia may provide additional prognostic information beyond conventional clinical risk factors, although the incremental prognostic findings were exploratory and require further validation. The clinical utility of sarcopenia assessment for mortality risk stratification remains to be established.

## Introduction

Sarcopenia, defined as an age-related decline in skeletal muscle mass, strength, and physical performance, is increasingly recognized as a major component of physical frailty among middle-aged and older adults [1]. Accumulating evidence has demonstrated that sarcopenia is associated with an increased risk of cardiovascular disease (CVD), cancer, disability, and all-cause mortality [2], [3], [4], [5]. In nephrology, sarcopenia is considered a manifestation of protein-energy wasting and contributes to the accelerated aging phenotype frequently observed in individuals with chronic kidney disease (CKD) [6], 7]. The pathophysiology of sarcopenia in CKD is complex and may be exacerbated by chronic inflammation, malnutrition, oxidative stress, metabolic disturbances, and physical inactivity [8], 9].

Sarcopenia is highly prevalent among community-dwelling middle-aged and older adults and is associated with adverse health outcomes, including increased mortality risk [10]. Previous studies have shown that sarcopenia predicts all-cause mortality in various populations, including older adults, patients with cirrhosis, nonalcoholic fatty liver disease, and those receiving dialysis [11], [12], [13], [14]. However, existing evidence has primarily focused on Western populations or individuals with advanced disease states, whereas the prognostic significance of sarcopenia in earlier stages of CKD remains insufficiently understood. Current evidence has predominantly focused on Western populations or on individuals with NAFLD, cirrhosis, or end-stage kidney disease.

Given that sarcopenia can be assessed using simple, non-invasive, and potentially modifiable measures, early identification may provide opportunities for targeted interventions, such as exercise and lifestyle modification [15]. Therefore, this study aimed to investigate the association between sarcopenia and all-cause mortality among Chinese community-dwelling middle-aged and older adults with an eGFR of 15–89 mL/min/1.73 m^2^ and to evaluate its incremental prognostic value beyond conventional clinical risk factors.

## Materials and methods

### Study population

The China Health and Retirement Longitudinal Study (CHARLS) is an ongoing nationwide prospective cohort study of Chinese adults aged 45 years and older designed to investigate aging-related health, socioeconomic, and functional outcomes. Detailed information regarding the study design and data collection procedures has been published previously [16]. Briefly, the baseline survey was conducted in 2011 and enrolled approximately 17,500 participants from 10,000 households across 150 counties/districts and 450 villages or urban communities throughout China. Follow-up surveys were conducted in, 2015, and 2018.

For the present study, we included community-dwelling middle-aged and older adults aged 45–85 years with an eGFR of 15–89 mL/min/1.73 m^2^, calculated using the Chronic Kidney Disease Epidemiology Collaboration (CKD-EPI) equation. Because albuminuria and other markers of kidney damage were not available for defining CKD in the present analysis, participants with an eGFR of 60–89 mL/min/1.73 m^2^ could not be confirmed to have CKD stage 2 solely on the basis of eGFR. Therefore, the study population was operationally defined using eGFR criteria rather than the complete clinical criteria for CKD stages 2–4. Participants with missing sarcopenia assessments, missing laboratory data, or unavailable survival or sex information were excluded. After applying the exclusion criteria, a total of 2,526 participants were included in the final analysis (Figure 1).

**Figure 1:**
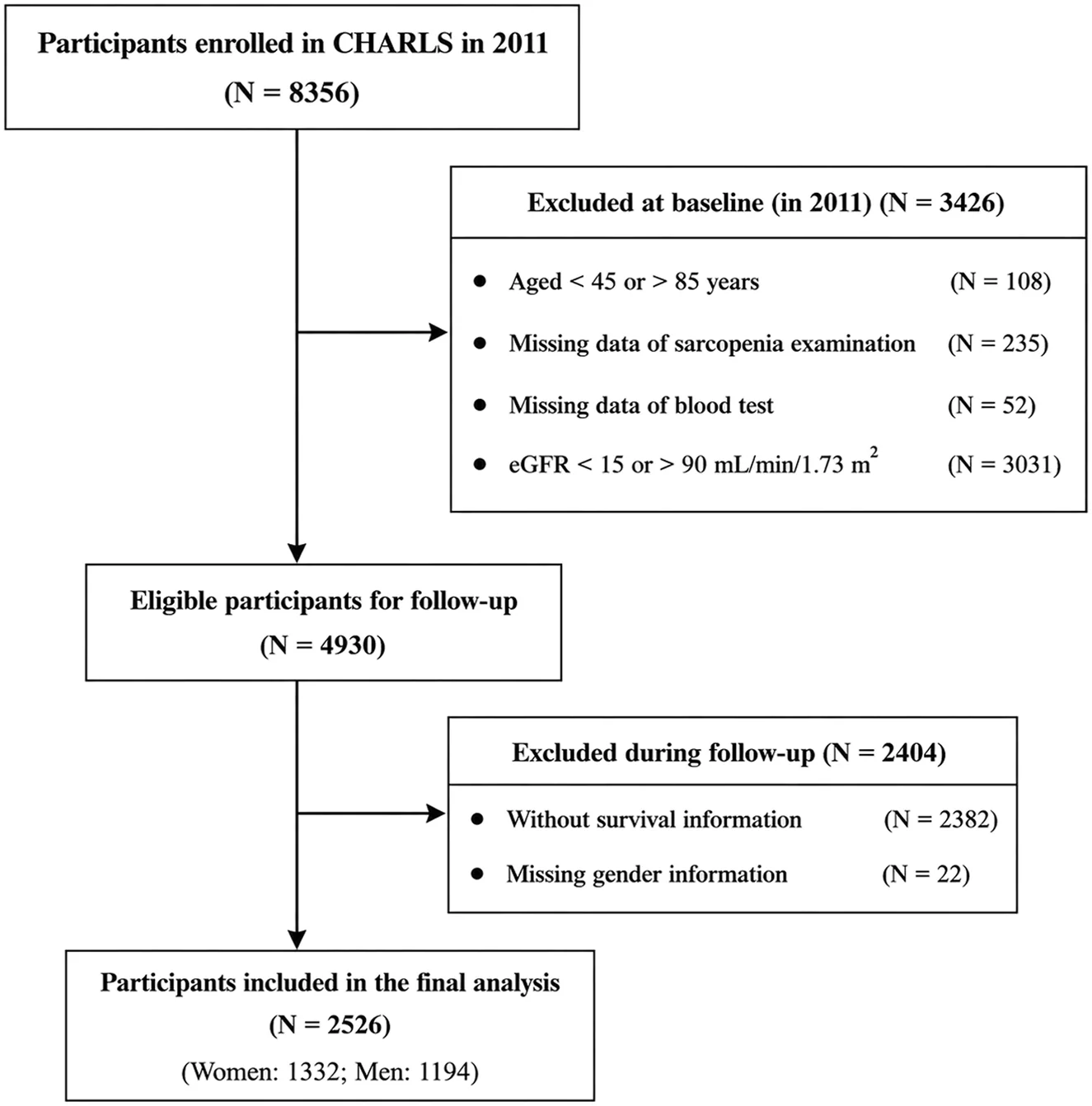
Patient flow diagram.

The CHARLS study protocol was approved by the Biomedical Ethics Committee of Peking University and was conducted in accordance with the Declaration of Helsinki (as revised in 2013). Written informed consent was obtained from all participants prior to enrollment.

### Definition of sarcopenia

Sarcopenia was operationally defined based on the framework of the Asian Working Group for Sarcopenia (AWGS) 2019 [17]. Because direct measurements of appendicular skeletal muscle mass (ASM) using dual-energy X-ray absorptiometry (DXA) or bioelectrical impedance analysis (BIA) were unavailable in CHARLS, ASM was estimated using a validated anthropometric equation [18]. Sex-specific cutoffs for low muscle mass were defined as the lowest 20 % of the ASM/height^2^ (kg/m^2^) distribution in the present study population, corresponding to <8.04 kg/m^2^ for men and <6.58 kg/m^2^ for women.

Muscle strength was assessed using handgrip strength, with low muscle strength defined as <28 kg in men or <18 kg in women. Grip strength was measured twice, and the highest average value was recorded. Physical performance was assessed using the 5-time chair stand test, with a completion time of ≥12 s considered poor physical performance. Sarcopenia was defined as low muscle mass accompanied by either low muscle strength or poor physical performance. Thus, the present study represents an operationalized/modified implementation of the AWGS 2019 framework, rather than a direct application of the standard AWGS 2019 muscle-mass thresholds.

### Assessment of covariates

Potential confounding variables included demographic characteristics, lifestyle factors, comorbidities, anthropometric measurements, laboratory parameters, and medication use.

Demographic characteristics included age, sex, educational attainment (0, 1–6, 7–9, 10–12, and >12 years), urban or rural residence, marital status (married/cohabiting or other), and ethnicity (Han or other). Lifestyle factors included smoking status (never, former, or current smoker) and alcohol consumption (never, former, or current drinker).

Comorbidities included hypertension, diabetes mellitus, dyslipidemia, and cardiovascular disease (CVD). CVD was defined as a self-reported history of angina, coronary heart disease, myocardial infarction, congestive heart failure, stroke, or other cardiovascular conditions. Hypertension was defined as systolic blood pressure ≥140 mmHg, diastolic blood pressure ≥90 mmHg, self-reported physician diagnosis, or use of antihypertensive medications [19]. Diabetes mellitus was defined as hemoglobin A1c (HbA1c) ≥6.5 %, self-reported physician diagnosis, or use of antidiabetic medications [20].

Anthropometric measurements included waist circumference (WC) and body mass index (BMI). Laboratory variables included fasting glucose, hemoglobin A1c (HbA1c), total cholesterol (TC), triglycerides (TG), high-density lipoprotein cholesterol (HDL-C), low-density lipoprotein cholesterol (LDL-C), serum uric acid (SUA), high-sensitivity C-reactive protein (hs-CRP), hemoglobin, serum creatinine, and estimated glomerular filtration rate (eGFR).

Medication use included antihyperglycemic, antihypertensive, and lipid-lowering medications.

### All-cause mortality

Vital status and dates of death were obtained from follow-up surveys conducted in 2013, 2015, and 2018. Follow-up time was calculated from the baseline assessment in 2011 to the date of death or the last available follow-up, whichever occurred first. Participants without survival information at the end of follow-up were excluded from the analysis (Figure 1).

### Statistical analyses

Baseline characteristics stratified by sex-specific sarcopenia status are presented as means ± standard deviations (SD), medians (interquartile ranges [IQR]), or percentages, as appropriate. Differences between groups were compared using Student’s t-tests or Wilcoxon rank-sum tests for continuous variables and chi-square tests or Fisher’s exact tests for categorical variables.

Cox proportional hazards regression models were used to evaluate the associations between sarcopenia (and its individual components) and all-cause mortality. Three models were sequentially adjusted. Model 1 was adjusted for age. Model 2 was additionally adjusted for education level, body mass index (BMI), hypertension, lipid profiles, estimated glomerular filtration rate (eGFR), high-sensitivity C-reactive protein (hs-CRP), smoking status, and alcohol consumption. Model 3 was further adjusted for antihyperglycemic, antihypertensive, and lipid-lowering medication use. Pre-specified subgroup analyses were conducted according to age, hypertension, diabetes, dyslipidemia, BMI, kidney function (eGFR), and hyperuricemia. Hazard ratios (HRs) and 95 % confidence intervals (CIs) were reported.

The incremental prognostic value of sarcopenia was evaluated by comparing a clinical variable-based Cox model with an extended Cox model that additionally included sarcopenia. For each participant, 5-year mortality risk predictions were derived from the Cox proportional hazards models using the Breslow estimator of the baseline hazard. Time-dependent receiver operating characteristic (ROC) analysis was used to account for censored survival data, and the time-dependent area under the curve (AUC) was calculated at the 5-year prediction horizon using the R package *survivalROC*. The integrated discrimination improvement (IDI) and continuous net reclassification improvement (NRI) were calculated by comparing the model-derived 5-year risk predictions from the two Cox models using the R package *PredictABEL*. These analyses were exploratory and were intended to assess whether sarcopenia provided incremental prognostic information beyond conventional clinical risk factors rather than to develop a formal prediction model.

Additional exploratory analyses were performed using Cox proportional hazards models to examine the associations between poor physical performance, defined as a 5-time chair stand test ≥12 s, and all-cause mortality in women overall and across prespecified subgroups. Cox regression analyses and other statistical analyses were performed using SAS version 9.4 (SAS Institute Inc., Cary, NC, USA), while time-dependent ROC, IDI, and continuous NRI analyses were performed using R with the *survivalROC* and *PredictABEL* packages.

## Results

### Patient characteristics

The baseline survey was conducted in 2011, with follow-up assessments performed in 2013, 2015, and 2018. A total of 3,426 participants were excluded for the following reasons: 108 were outside the predefined age range (45–85 years), 235 had missing sarcopenia assessment data, 52 lacked laboratory measurements, and 3,031 had an estimated glomerular filtration rate (eGFR) >90 mL/min/1.73 m^2^. In addition, 2,382 participants without available survival information were excluded. Ultimately, 2,526 participants were included in the final analysis, comprising 1,332 women and 1,194 men (Figure 1). During follow-up, 127 of 1,332 women (9.5 %) and 112 of 1,194 men (9.3 %) died. A comparison of baseline characteristics between the included participants and those excluded because of missing survival information is presented in Supplementary Table S1.

Among women, participants with sarcopenia were significantly older than those without sarcopenia (64.6 ± 8.0 vs. 61.3 ± 8.2 years). They also had higher rates of hypertension and antihypertensive medication use, lower proportions of being married and having a senior school education, lower levels of serum uric acid (SUA), low-density lipoprotein cholesterol (LDL-C), creatinine, and eGFR, and higher levels of total cholesterol.

Among men, participants with sarcopenia were also significantly older than those without sarcopenia (66.3 ± 8.4 vs. 63.4 ± 8.4 years). They had higher rates of antihyperglycemic medication use and lower proportions of being married, having a senior school education, and alcohol consumption, as well as lower levels of SUA and eGFR (Table 1). The prevalence of sarcopenia across eGFR categories, stratified by sex, is shown in Supplementary Table S2.

**Table 1:** Baseline characteristics according to sarcopenia status, stratified by sex.

Variable	Men non-sarcopenia (n=859)	Men sarcopenia (n=335)	p-Value	Women non-sarcopenia (n=781)	Women sarcopenia (n=551)	p-Value
**Death, n (%)**	82 (9.5)	30 (9.0)	0.825	59 (7.6)	68 (12.3)	0.004

**Demographics and lifestyle**						

Age, years	63.4 ± 8.4	66.3 ± 8.4	<0.001	61.3 ± 8.2	64.6 ± 8.0	<0.001
Married/cohabiting, n (%)	728 (84.8)	265 (79.1)	0.019	562 (72.0)	334 (60.6)	<0.001
Han ethnicity, n (%)	816 (95.0)	308 (91.9)	0.044	723 (92.6)	507 (92.0)	0.706
Agricultural residence, n (%)	689 (80.2)	282 (84.4)	0.093	691 (88.5)	496 (90.0)	0.374

**Education level, n (%)**						

0 years	100 (11.6)	63 (18.8)		349 (44.7)	287 (52.1)	
1–6 years	453 (52.7)	188 (56.1)		315 (40.3)	218 (39.6)	
7–9 years	198 (23.1)	64 (19.1)		81 (10.4)	30 (5.4)	
10–12 years	68 (7.9)	14 (4.2)		28 (3.6)	13 (2.4)	
>12 years	40 (4.7)	6 (1.8)	<0.001	8 (1.0)	3 (0.5)	<0.001

**Clinical characteristics**						

Systolic BP, mmHg	131.1 ± 21.2	130.7 ± 21.1	0.733	131.5 ± 22.6	135.8 ± 23.2	<0.001
Diastolic BP, mmHg	75.2 ± 12.3	74.7 ± 12.4	0.521	74.9 ± 11.2	76.0 ± 11.9	0.092
HTN, n (%)	353 (41.1)	156 (46.6)	0.086	342 (43.8)	281 (51.0)	0.009
Diabetes, n (%)	58 (6.8)	23 (6.9)	0.944	57 (7.3)	52 (9.4)	0.161
Dyslipidemia, n (%)	48 (5.6)	17 (5.1)	0.722	78 (10.0)	53 (9.6)	0.818

**Smoking status**			0.586			0.205

Never	214 (24.9)	77 (23.3)		711 (91.2)	490 (89.3)	
Former	131 (15.3)	58 (17.6)		15 (1.9)	19 (3.5)	
Current	513 (59.8)	195 (59.1)		54 (6.9)	40 (7.3)	

**Drinking status**			0.025			0.530

Never	394 (45.9)	180 (53.7)		691 (88.5)	494 (89.7)	
Former	96 (11.2)	25 (7.5)		40 (5.1)	21 (3.8)	
Current	369 (43.0)	130 (38.8)		50 (6.4)	36 (6.5)	

**Physical examination**						

BMI, kg/m^2^			0.690			0.394
Lean (<18.5)	64 (7.5)	28 (8.4)		59 (7.6)	39 (7.2)	
Normal (18.5–23.9)	515 (60.2)	202 (60.7)		374 (48.1)	255 (46.8)	
Overweight (24–27.9)	215 (25.2)	75 (22.5)		238 (30.6)	158 (29)	
Obesity (≥28.0)	61 (7.1)	28 (8.4)		106 (13.6)	93 (17.1)	

**Waist circumference, cm**			0.160			0.108

≥90 for male or 85 for female	356 (41.5)	154 (46.0)		517 (66.4)	388 (70.6)	

**Inflammation, glucose, lipids and kidney function**						

Has-CRP, mg/L, median (IQR)	1.1 (0.6–2.2)	1.2 (0.6–2.3)	0.307	1.1 (0.6–2.1)	1.1 (0.6–2.3)	0.670
SUA, mg/dL	5.1 (4.3–5.9)	4.8 (4.2–5.8)	0.015	4.2 (3.6–4.9)	4.1 (3.5–4.7)	0.003
Total cholesterol, mg/dL	183.6 (161.2–207.6)	183.6 (162.4–204.5)	0.357	199.1 (177.8–225)	191.8 (169.3–219.2)	0.007
HDL cholesterol, mg/dL	49.1 (40.2–60.7)	45.6 (38.7–55.7)	0.055	51.8 (42.5–61.5)	50.3 (40.2–60.3)	0.570
LDL cholesterol, mg/dL	110.2 (89.3–132.2)	112.1 (91.2–134.2)	0.909	122.2 (100.3–146.1)	114.8 (93.6–139.9)	0.001
Triglycerides, mg/dL	93.8 (68.1–138.9)	98.2 (72.6–138.1)	0.529	111.5 (80.5–154.9)	115.9 (83.2–168.1)	0.835
HbA1c, %	5.1 (4.9–5.4)	5.1 (4.9–5.4)	0.556	5.2 (4.9–5.5)	5.2 (4.9–5.5)	0.850
Fasting glucose, mg/dL	101.9 (94.1–112.1)	102.4 (94.5–113.9)	0.886	102.6 (94.1–112)	102.6 (95.0–113.2)	0.818
Creatinine, mg/dL	0.927 (0.825–1.040)	0.904 (0.802–1.028)	<0.001	0.757 (0.678–0.836)	0.735 (0.655–0.825)	<0.001
Hemoglobin, g/dL	15 (13.8–16.2)	15 (13.9–16.2)	0.198	13.7 (12.6–14.7)	13.6 (12.6–14.7)	0.170
eGFR, mL/min/L.73 m^2^	78.0 (68.4–84.6)	74.6 (67.6–82.3)	<0.001	79.8 (71.3–85.3)	78.7 (70.0–84.7)	0.009

**Medication use**						

Antihyperglycemic drugs	27 (3.5)	24 (4.4)	0.012	25 (2.9)	8 (2.4)	0.126
Antihypertensive drugs	162 (18.9)	80 (23.9)	0.053	161 (20.6)	173 (31.4)	<0.001
Lipid-lowering drugs	32 (3.7)	24 (7.2)	0.621	36 (4.6)	36 (6.5)	0.400

Data are presented as mean ± SD, median (IQR), or n (%), as appropriate. p values were calculated using Student’s t-test or Wilcoxon rank-sum test for continuous variables and chi-square test or Fisher’s exact test for categorical variables. BMI, body mass index; BP, blood pressure; CRP, C-reactive protein; eGFR, estimated glomerular filtration rate; SUA, serum uric acid; HDL, high-density lipoprotein; HbA1c, Hemoglobin A1c; HTN, hypertension; LDL, low-density lipoprotein.

### Effects of sarcopenia and its components on all-cause mortality

Sarcopenia and poor physical performance were significantly associated with all-cause mortality among women, whereas no statistically significant associations were observed among men. After full adjustment, women with sarcopenia had an 82 % higher risk of all-cause mortality (HR, 1.82; 95 % CI, 1.26–2.63), while a 5-time chair stand test ≥12 s was associated with an 86 % higher mortality risk (HR, 1.86; 95 % CI, 1.29–2.68). No significant associations were observed in men (Table 2).

**Table 2:** Sex-specific associations of sarcopenia and its components with all-cause mortality (HRs and 95 % CIs).

	Men	p-Value	Women	p-Value
**Outcome**				
**Sarcopenia**	16.9 (30/1,779), per 1,000 person-years, n/N		22.9 (68/2,965), per 1,000 person-years, n/N	
**Non-sarcopenia**	17.2 (82/4,781)		14.2 (59/4,149)	
Model 1	0.97 (0.64–1.48)	0.883	1.69 (1.18–2.41)	0.004
Model 2	1.06 (0.69–1.60)	0.798	1.74 (1.21–2.50)	0.003
Model 3	1.05 (0.68–1.61)	0.825	1.82 (1.26–2.63)	0.001
**Muscle strength <28 kg for males, or <18 kg for females**	13.8 (15/1,087), per 1,000 person-years, n/N		20.6 (22/1,066), per 1,000 person-years, n/N	
**Muscle strength ≥28 kg for males, or ≥18 kg for females**	17.4 (97/5,561), per 1,000 person-years, n/N		17.4 (107/6,136), per 1,000 person-years, n/N	
Model 1	0.79 (0.45–1.38)	0.409	1.22 (0.76–1.95)	0.410
Model 2	0.79 (0.45–1.39)	0.407	1.15 (0.69–1.91)	0.593
Model 3	0.80 (0.45–1.42)	0.443	1.15 (0.69–1.91)	0.587
**ASM/Hit** ^**2**^ **<8.04 kg for males, or <6.58 kg for females**	12.6 (17/1,354), per 1,000 person-years, n/N		17.7 (26/1,467), per 1,000 person-years, n/N	
**ASM/Hit** ^**2**^ **≥8.04 kg for males, or ≥6.58 kg for females**	15.1 (95/5,255), per 1,000 person-years, n/N		18.0 (103/5,735), per 1,000 person-years, n/N	
Model 1	0.70 (0.41–1.18)	0.177	0.96 (0.62–1.50)	0.869
Model 2	0.62 (0.31–1.26)	0.188	1.05 (0.56–1.97)	0.877
Model 3	0.65 (0.32–1.31)	0.225	1.07 (0.57–2.01)	0.835
**5-Time chair stand test≥12 s**	16.7 (29/1,737), per 1,000 person-years, n/N		23.2 (67/2,888), per 1,000 person-years, n/N	
**5-Time chair stand test<12 s**	17.2 (83/4,823), per 1,000 person-years, n/N		14.2 (60/4,226), per 1,000 person-years, n/N	
Model 1	0.96 (0.62–1.47)	0.836	1.72 (1.21–2.46)	0.003
Model 2	1.05 (0.68–1.62)	0.834	1.77 (1.23–2.55)	0.002
Model 3	1.04 (0.67–1.60)	0.863	1.86 (1.29–2.68)	<0.001

Model 1: adjusted for age. Model 2: additionally adjusted for marital status, educational attainment, hypertension, BMI, TC, HDL, TG, Hemoglobin, eGFR, hs-CRP, smoking and drinking status. Model 3: further adjusted for antihyperglycemic drugs, antihypertensive drugs and lipid-lowering drugs. ASM, appendicular skeletal muscle mass; CI, confidence interval; Hit, height; HR, hazard ratio.

In prespecified subgroup analyses, numerically larger effect estimates for the association between sarcopenia and all-cause mortality were observed among women aged 45–60 years, those without hypertension or diabetes, those with BMI<24.0 kg/m^2^, those with an eGFR of 60–89 mL/min/1.73 m^2^, and those without hyperuricemia. However, none of the interaction tests reached statistical significance (all p for interaction >0.05), and statistically significant differences between these subgroups were not demonstrated. These findings should therefore be considered exploratory and interpreted cautiously (Table 3).

**Table 3:** Sex-specific subgroup analyses of the association between sarcopenia and all-cause mortality.

		Men						Women			
		Crude incidence rate per 1,000 person-years (events/person-years)	HR (95 % CI)	p-Value	p for interaction			Crude incidence rate per 1,000 person-years (events/person-years)	HR (95 % CI)	p-Value	p for interaction
Age >60 years (n=775)	Sarcopenia	15.1 (19/1,262)	0.89 (0.51–1.54)	0.677	0.218	Age >60 years (n=785)	Sarcopenia	20.8 (42/2,024)	1.53 (0.95–2.47)	0.083	0.566
	Non-sarcopenia	16.9 (50/2,964)	1.00 (ref)				Non-sarcopenia	15.0 (33/2,195)	1.00 (ref)		
Age 45–60 years (n=419)	Sarcopenia	21.3 (11/517)	1.28 (0.62–2.64)	0.502		Age 45–60 years (n=547)	Sarcopenia	27.6 (26/941)	2.31 (1.3–4.11)	0.005	
	Non-sarcopenia	17.6 (32/1,817)	1.00 (ref)				Non-sarcopenia	13.3 (26/1,954)	1.00 (ref)		
HTN (n=509)	Sarcopenia	16.7 (14/838)	0.98 (0.51–1.89)	0.959	0.948	HTN (n=623)	Sarcopenia	19.3 (30/1,552)	1.30 (0.77–2.21)	0.325	0.231
	Non-sarcopenia	16.6 (33/1,983)	1.00 (ref)				Non-sarcopenia	16.8 (31/1,844)	1.00 (ref)		
Non-HTN (n=685)	Sarcopenia	17.0 (16/941)	1.00 (0.55–1.82)	0.989		Non-HTN (n=709)	Sarcopenia	26.9 (38/1,413)	2.35 (1.4–3.94)	0.001	
	Non-sarcopenia	17.5 (49/2,798)	1.00 (ref)				Non-sarcopenia	12.1 (28/2,305)	1.00 (ref)		
Diabetes (n=81)	Sarcopenia	7.8 (1/128)	N. A	N. A	0.425	Diabetes (n=109)	Sarcopenia	25.0 (7/280)	1.10 (0.28–4.31)	0.893	0.845
	Non-sarcopenia	3.0 (1/338)					Non-sarcopenia	18.0 (5/278)	1.00 (ref)		
Non-diabetes (n=1,113)	Sarcopenia	17.6 (29/1,651)	1.04 (0.67–1.60)	0.878		Non-diabetes (n=1,223)	Sarcopenia	22.7 (61/2,685)	1.84 (1.25–2.71)	0.002	
	Non-sarcopenia	18.2 (81/4,443)	1.00 (ref)				Non-sarcopenia	13.9 (54/3,871)	1.00 (ref)		
BMI≥24.0 kg/m^2^ (n=379)	Sarcopenia	12.3 (7/568)	0.73 (0.31–1.75)	0.479	0.318	BMI≥24.0 kg/m^2^ (n=595)	Sarcopenia	21.1 (28/1,327)	1.54 (0.89–2.66)		0.122
	Non-sarcopenia	16.1 (25/1,553)	1.00 (ref)				Non-sarcopenia	15.4 (29/1,883)	1.00 (ref)		
BMI<24.0 kg/m^2^ (n=809)	Sarcopenia	19.2 (23/1,197)	1.24 (0.75–2.05)	0.410		BMI<24.0 kg/m^2^ (n=727)	Sarcopenia	23.6 (38/1,610)	2.07 (1.26–3.41)	0.004	
	Non-sarcopenia	17.7 (57/3,214)	1.00 (ref)				Non-sarcopenia	13.4 (30/2,245)	1.00 (ref)		
eGFR 15–60 mL/min/1.73 m² (n=142)	Sarcopenia	10.1 (2/198)	0.95 (0.13–7.05)	0.963	0.402	eGFR 15–60 mL/min/1.73 m² (n=123)	Sarcopenia	8.5 (3/352)	3.14 (0.1–103.01)	0.520	0.091
	Non-sarcopenia	17.4 (9/518)	1.00 (ref)				Non-sarcopenia	10.0 (3/301)	1.00 (ref)		
eGFR 60–89 mL/min/1.73 m² (n=1,052)	Sarcopenia	17.7 (28/1,581)	1.10 (0.70–1.72)	0.687		eGFR 60–89 mL/min/1.73 m² (n=1,209)	Sarcopenia	24.9 (65/2,613)	1.88 (1.29–2.73)	0.001	
	Non-sarcopenia	17.1 (73/4,263)	1.00 (ref)				Non-sarcopenia	14.6 (56/3,848)	1.00 (ref)		
HUA (n=98)	Sarcopenia	20.4 (2/98)	1.34 (0.11–16.91)	0.819	0.794	HUA (n=98)	Sarcopenia	11.8 (2/170)	0.82 (0.06–10.59)	0.890	0.351
	Non-sarcopenia	14.5 (6/414)	1.00 (ref)				Non-sarcopenia	24.7 (7/283)	1.00 (ref)		
Non-HUA (n=1,096)	Sarcopenia	16.7 (28/1,681)	1.02 (0.65–1.59)	0.93		Non-HUA (n=1,234)	Sarcopenia	23.6 (66/2,795)	2.01 (1.38–2.95)	<0.001	
	Non-sarcopenia	17.4 (76/4,367)	1.00 (ref)				Non-sarcopenia	13.5 (52/3,866)	1.00 (ref)		

HRs were adjusted for age, marital status, educational attainment, hypertension, BMI, TC, HDL, TG, hemoglobin, eGFR, hs-CRP, smoking, drinking status, antihyperglycemic drugs, and antihypertensive drugs. These subgroup analyses were exploratory and should be interpreted cautiously because multiple comparisons were performed and no significant interaction effects were observed. BMI, body mass index; CKD, chronic kidney disease; CI, confidence interval; HR, hazard ratio; HTN, hypertension; HUA, hyperuricemia.

### Additive value of sarcopenia for prediction of all-cause mortality among women

The addition of sarcopenia to the clinical variable-based model was associated with a modest improvement in mortality risk prediction in women, as evaluated using time-dependent ROC analysis at the 5-year prediction horizon. The time-dependent AUC increased from 0.615 (95 % CI, 0.561–0.670) for the clinical variable-based model to 0.650 (95 % CI, 0.599–0.700) for the model additionally incorporating sarcopenia. The IDI was 0.008 (95 % CI, 0.003–0.014; p=0.006), whereas the continuous NRI was 0.236 (95 % CI, −0.055–0.417; p=0.090). These findings suggest that sarcopenia may provide modest incremental prognostic information beyond conventional clinical risk factors in women (Table 4).

**Table 4:** Incremental prognostic value of sarcopenia for all-cause mortality in women.

	5-year time-dependent AUC (95 % CI)	NRI (95 % CI)	p-Value	IDI (95 % CI)	p-Value
Basic model^a^	0.615 (0.561–0.670)				
Basic model + “sarcopenia”	0.650 (0.599–0.700)	0.236 (−0.055–0.417)	0.090	0.008 (0.003–0.014)	0.006

^a^Clinical model included age, educational attainment, hypertension, BMI, lipid profiles, hemoglobin, eGFR, hs-CRP, smoking status, alcohol consumption, and antihypertensive medication use. AUC, area under curve; NRI, net reclassification index; IDI, integrated discrimination improvement.

### Exploratory analyses

Exploratory analyses showed numerically larger effect estimates in some subgroups, including women aged 45–60 years, those without hypertension or diabetes, those with BMI<24.0 kg/m^2^, those with an eGFR of 60–89 mL/min/1.73 m^2^, and those without hyperuricemia. However, none of the interaction tests reached statistical significance (all p for interaction >0.05), and these findings should therefore be interpreted cautiously (Table 5).

**Table 5:** Exploratory subgroup analyses of the association between poor physical performance (5-time chair stand test ≥12 s) and all-cause mortality in women.

Subgroup	Crude incidence rate per 1,000 person-years (events/person-years)	HR (95 % CI)	p-Value	p for interaction
Age >60 years	21.0 (42/2,003)	1.58 (0.98–2.54)	0.062	0.567
Age 45–60 years	28.3 (25/885)	2.42 (1.35–4.33)	0.003	
HTN	19.9 (30/1,510)	1.44 (0.84–2.45)	0.181	0.289
Non-HTN	26.9 (37/1,378)	2.33 (1.39–3.92)	0.001	
Diabetes	26.3 (7/266)	1.12 (0.28–4.47)	0.874	0.755
Non-diabetes	22.9 (60/2,622)	1.88 (1.27–2.77)	0.002	
BMI ≥24.0 kg/m^2^	21.5 (27/1,250)	1.62 (0.93–2.81)	0.089	0.639
BMI <24.0 kg/m^2^	13.4 (38/1,610)	2.07 (1.26–3.41)	0.004	
eGFR 15–<60 mL/min/1.73 m^2^	8.5 (3/352)	3.14 (0.1–103.01)	0.520	0.088
eGFR 60–89 mL/min/1.73 m^2^	25.2 (64/2,536)	1.94 (1.33–2.82)	<0.001	
HUA	11.7 (2/170)	0.82 (0.06–10.59)	0.880	0.341
Non-HUA	23.9 (65/2,718)	2.07 (1.41–3.04)	<0.001	

Poor physical performance was defined as a 5-time chair stand test ≥12 s. HRs compare participants with poor physical performance (≥12 s) with those with better physical performance (<12 s). These subgroup analyses were exploratory and should be interpreted cautiously because multiple comparisons were performed and no statistically significant interaction effects were observed. BMI, body mass index; eGFR, estimated glomerular filtration rate; HR, hazard ratio; HTN, assess; HUA, hyperuricemia.

In exploratory analyses, numerically greater improvements in discrimination and reclassification were observed in several female subgroups, including those without hypertension, without diabetes, with BMI<24 kg/m^2^, and with eGFR 60–89 mL/min/1.73 m^2^. Given the multiple subgroup comparisons and the exploratory nature of these analyses, these findings should be interpreted cautiously and considered hypothesis-generating (Supplementary Table S3).

## Discussion

In this nationwide cohort of Chinese community-dwelling middle-aged and older adults with an eGFR of 15–89 mL/min/1.73 m^2^, we found that sarcopenia was independently associated with an increased risk of all-cause mortality in women, whereas no statistically significant association was observed in men.f Furthermore, poor physical performance, assessed by the 5-time chair stand test, emerged as an independent predictor of mortality among women. The addition of sarcopenia to a model containing conventional clinical risk factors modestly improved mortality risk prediction, suggesting that sarcopenia may provide incremental prognostic information beyond conventional clinical risk factors.

The relationship between sarcopenia and adverse outcomes has been demonstrated in several chronic diseases, including cirrhosis, diabetes, and advanced kidney disease. Previous studies have shown that sarcopenia is associated with increased mortality among patients with CKD, including those receiving hemodialysis, and peritoneal dialysis [21], [22], [23]. However, evidence regarding earlier stages of CKD remains limited. Our findings extend the current literature by demonstrating that the prognostic significance of sarcopenia may already be present in individuals with mild-to-moderate kidney dysfunction living in the community. Because sarcopenia can be assessed using simple, inexpensive, and widely available tools, routine evaluation may facilitate early risk stratification.

An important finding of our study was the apparent sex-specific association between sarcopenia and mortality. Although the underlying mechanisms remain incompletely understood, several biological pathways may explain the observed association. Sarcopenia and adverse clinical outcomes may share several biological mechanisms related to aging, chronic low-grade inflammation, metabolic dysregulation, endocrine alterations, mitochondrial dysfunction, cellular senescence, and impaired energy-sensing pathways such as AMP-activated protein kinase (AMPK) signaling [24]. Epidemiological studies have suggested an association between lower testosterone levels and reduced muscle mass or function [25]. Skeletal muscle homeostasis is regulated by multiple interconnected pathways, including hormonal regulation, mitochondrial function, inflammatory signaling, and age-related metabolic alterations [26], 27]. Declining estrogen levels after menopause, altered endocrine regulation, chronic low-grade inflammation, and reduced physical reserve may render women more susceptible to the adverse consequences of sarcopenia. In contrast, no statistically significant association was observed in men. However, this finding should not be interpreted as evidence that sarcopenia is not prognostic in men or that its effect is restricted to women. The lack of a statistically significant association in men may reflect limited statistical power, residual confounding, or potential biological differences between sexes. Moreover, the number of mortality events was relatively limited in both women and men. Therefore, these sex-specific findings should be interpreted cautiously and require confirmation in larger, independent cohorts, as the underlying mechanisms cannot be fully elucidated in the present observational study.

Understanding the sex-specific differences observed in sarcopenia may provide additional insights into its pathophysiological significance and facilitate the development of more individualized risk assessment strategies. Given the growing interest in precision medicine, identifying simple and clinically accessible biomarkers to improve prognostic evaluation has become increasingly important. Previous studies have shown that incorporating sarcopenia into conventional risk models may improve the prediction of adverse outcomes, including mortality, coronary heart disease, and cognitive impairment [28], [29], [30], [31]. Consistent with these findings, our results suggest that sarcopenia may provide modest incremental prognostic information beyond traditional clinical risk factors, among women in our cohort with an eGFR of 15–89 mL/min/1.73 m^2^. However, the improvement in predictive performance was modest, despite a statistically significant improvement in IDI, and the continuous NRI did not reach statistical significance. Therefore, the incremental clinical utility of incorporating sarcopenia into routine risk assessment remains uncertain and should be confirmed through external validation in independent cohorts before its incorporation into routine clinical practice.

We also observed that poor physical performance, rather than low muscle mass or low muscle strength alone, was independently associated with mortality among women. Physical performance likely reflects multiple dimensions of physiological aging, including neuromuscular coordination, balance, mobility, overall functional status, and broader physiological reserve [32], [33], [34]. Because impaired physical performance is potentially modifiable through exercise and rehabilitation, early identification may provide opportunities to prevent functional decline and improve long-term outcomes.

Exploratory subgroup analyses showed numerically larger effect estimates in several female subgroups, including those without hypertension or diabetes, with BMI<24 kg/m^2^, with eGFR 60–89 mL/min/1.73 m^2^, and without hyperuricemia. However, none of the interaction tests reached statistical significance, and multiple subgroup analyses were performed. Therefore, these findings should be considered exploratory and hypothesis-generating rather than evidence of statistically significant effect modification.

Our study has several strengths, including the use of a large nationwide cohort with standardized data collection and long-term follow-up. Nevertheless, several limitations should be acknowledged. First, a substantial proportion of participants were excluded because of missing data or unavailable survival information, which may have introduced attrition and selection bias and limited the generalizability of our findings. Although baseline characteristics were compared between the included participants and those excluded because of missing survival information (Supplementary Table 1), the large proportion of participants with unavailable survival information means that residual selection bias cannot be excluded. This substantial attrition may also have affected the representativeness of the analytic cohort and the observed mortality estimates. Second, the observational design precludes causal inference, and residual confounding cannot be entirely excluded despite extensive adjustment for potential confounders. Third, CKD status was operationally defined using eGFR rather than a comprehensive definition incorporating markers of kidney damage. In particular, participants with an eGFR of 60–89 mL/min/1.73 m^2^ may not have had CKD in the absence of albuminuria or other evidence of kidney damage. Because albuminuria and other markers of kidney damage were not available for CKD classification in the present analysis, some participants with preserved or mildly reduced eGFR may have been misclassified as having CKD stage 2. This limitation is particularly relevant because most participants in our cohort had an eGFR of 60–89 mL/min/1.73 m^2^. Therefore, our findings should be interpreted as applying to an eGFR-defined population rather than a population with definitively established CKD stages 2–4. Fourth, direct measurements of muscle mass, such as dual-energy X-ray absorptiometry (DXA) or bioelectrical impedance analysis (BIA), were unavailable in CHARLS. Consequently, ASM was estimated using a validated anthropometric equation, and sex-specific cutoffs were derived from the lowest 20 % of the ASM/height^2^ distribution in the study population, an approach that has been used in previous studies [18]. Therefore, our approach represents an operationalized/modified implementation of the AWGS 2019 framework rather than a direct application of the standard AWGS 2019 muscle-mass thresholds. Nevertheless, this indirect approach may have introduced misclassification of low muscle mass and sarcopenia status. Finally, cause-specific mortality data were unavailable; therefore, we could not examine cardiovascular-, kidney-, or other cause-specific mortality. Future prospective studies incorporating direct measurements of muscle mass, comprehensive assessment of kidney damage, and external validation in independent cohorts are warranted to confirm and extend our findings.

## Conclusions

In this nationwide cohort of community-dwelling adults with an eGFR of 15–89 mL/min/1.73 m^2^, sarcopenia was associated with an increased risk of all-cause mortality among women. Sarcopenia may provide additional prognostic information beyond conventional clinical risk factors and may facilitate risk stratification in this population. Prospective studies incorporating direct measurements of muscle mass and external validation in independent cohorts are warranted to confirm these findings and further clarify whether sex-related differences in the prognostic significance of sarcopenia exist.

## Supplementary Material

Supplementary Material
