# Supplementary material for: The role of sarcopenia in all-cause mortality prediction among community-dwelling adults with eGFR 15–89 mL/min/1.73 m2
Source: Open Med (Wars). 2026 Oct 22;21(1):20261531. doi: 10.1515/med-2026-1531 (PMC13686430; doi:10.1515/med-2026-1531)
Supplement: Supplementary file 1 [file j_med-2026-1531_suppl_001.docx]

**S****upplementary Table S1. Comparison of baseline characteristics between the included participants and those excluded because of missing survival data**

| **Characteristic** | **Included participants** | **Excluded participants** | P value |
| --- | --- | --- | --- |
| **N** | 2526 | 2382 |  |
| **Demographics, Cardiovascular history and lifestyle** |  |  |  |
| Age, years | 63.4±8.4 | 58.8±9.2 | <0.001 |
| Male, n (%) | 1194(47.3) | 1061 (44.5) | 0.059 |
| Married/cohabitating, n (%) | 1889 (74.8) | 1829 (76.8) | 0.109 |
| Han ethnicity, n (%) | 2354 (93.2) | 2211 (92.8) | 0.652 |
| Agricultural residence, n (%) | 2158 (85.4) | 2059 (86.4) | 0.33 |
| **Education, years** |  |  | 0.835 |
| 0 years | 799 (31.6) | 744 (31.2) |  |
| 1–6 years | 1174 (46.5) | 1132 (47.5) |  |
| 7–9 years | 373 (14.8) | 344 (14.4) |  |
| 10–12 years | 123 (4.9) | 118 (5.0) |  |
| >12 years | 57 (2.3) | 44 (1.8) |  |
| **HTN, n (%)** | 1132 (44.8) | 869 (36.5) | <0.001 |
| **Diabetes, n (%)** | 190 (7.5) | 137 (5.8) | 0.015 |
| **Smoking status** |  |  | 0.002 |
| Never | 1492 (59.1) | 1519 (63.8) |  |
| Former | 223 (8.8) | 169 (7.1) |  |
| Current | 802 (31.7) | 684 (28.7) |  |
| **Drinking status** |  |  |  |
| Never | 1759 (69.6) | 1606 (67.4) | 0.229 |
| Former | 182 (7.2) | 192 (8.1) |  |
| Current | 585 (23.2) | 583 (24.5) |  |
| **Physical Examination** |  |  |  |
| **BMI, kg/m2** |  |  | 0.109 |
| Lean (<18.5) | 190 (7.5) | 164 (6.9) |  |
| Normal (18.5–23.9) | 1346 (53.3) | 1309 (55.0) |  |
| Overweight (24–27.9) | 686 (27.2) | 634 (26.6) |  |
| Obesity (≥28.0) | 288 (11.4) | 223 (9.4) |  |
| **Waist circumference, cm** |  |  |  |
| ≥90 for male or 85 for female | 1415 (56.0) | 1223 (51.3) | 0.001 |
| **Inflammation, glucose, lipids**  **and renal function** |  |  |  |
| hs-CRP, mg/L, median (IQR) | 1.12 (0.59–2.20) | 0.96 (0.53–2.09) | <0.001 |
| SUA, mg/dL | 4.52 (3.80–5.40) | 4.30 (3.57–5.19) | <0.001 |
| Total cholesterol, mmol/L | 190.98 (167.40–215.34) | 189.43 (166.24–213.02) | 0.2375 |
| HDL cholesterol, mmol/L | 49.87 (40.59–60.70) | 50.64 (41.37–60.31) | 0.1993 |
| LDL cholesterol, mmol/L | 115.21 (93.94–138.40) | 112.11 (92.40–132.60) | <0.001 |
| Triglycerides, mmol/L | 103.54 (75.22–148.24) | 100.89 (72.57–146.25) | 0.1653 |
| HbA1c, % | 5.20 (4.90–5.40) | 5.20 (4.90–5.43) | 0.4549 |
| Creatinine, mg/dL | 0.81 (0.71–0.94) | 0.76 (0.66–0.88) | <0.001 |
| Hemoglobin, g/L | 14.20 (13.00–15.60) | 13.90 (12.80–15.30) | <0.001 |

**Supplementary Table S2. Prevalence of sarcopenia according to eGFR category and sex**

|  | **men** | | | |  | **women** | | | |
| --- | --- | --- | --- | --- | --- | --- | --- | --- | --- |
|  | **Sarcopenia** | **No sarcopenia** | | **P value** |  | **Sarcopenia** | **No sarcopenia** | | **P value** |
| eGFR, mL/ min/l.73m^2^ |  |  | | 0.001 |  |  |  | | <0.001 |
| ≥90 | 268 (21.7) | 967 (78.3) | |  |  | 494 (29.9) | 1157 (70.1) | |  |
| 60–89 | 297 (28.2) | 758 (71.9) | |  |  | 490 (40.4) | 723 (59.6) | |  |
| <60 | 38 (27.3) | 101 (72.7) | |  |  | 60 (50.4) | 59 (49.6) | |  |
|  | *P* _trend_ =0.001 | |  | |  | *P* _trend_ <0.001 | |  | |

Abbreviations: eGFR, estimated glomerular filtration rate.

**Supplementary Table S3. Incremental prognostic value of sarcopenia for all-cause mortality across prespecified subgroups among women**

|  | **AUC (95%CI)** | **NRI (95% CI)** | **P value** | **IDI (95% CI)** | **P value** |
| --- | --- | --- | --- | --- | --- |
| Age >60 years | 0.624(0.553–0.694) |  |  |  |  |
| Model1 | 0.637 (0.568–0.706) | 0.17 (-0.040–0.393) | 0.219 | 0.007 (0.001–0.014) | 0.005 |
| Model1+"sarcopenia" |  |  |  |  |  |
| Age 45–60 years | 0.672 (0.590–0.754) |  |  |  |  |
| Model1 | 0.727 (0.661–0.793) | 0.337 (0.023–0.651) | 0.016 | 0.007(-0.006–0.021) | 0.153 |
| Model1+"sarcopenia" | 0.624 (0.553–0.694) |  |  |  |  |
| HTN |  |  |  |  |  |
| Model1 | 0.672 (0.597–0.747) |  |  |  |  |
| Model1+"sarcopenia" | 0.669 (0.595–0.744) | 0.092(-0.170–0.354) | 0.509 | 0.002(-0.003–0.008) | 0.173 |
| Non-HTN |  |  |  |  |  |
| Model1 | 0.623 (0.555–0.692) |  |  |  |  |
| Model1+"sarcopenia" | 0.690 (0.629–0.751) | 0.366 (0.116–0.616) | 0.009 | 0.016 (0.005–0.026) | 0.006 |
| Dyslipidemia |  |  |  |  |  |
| Model1 | 0.863 (0.760–0.966) |  |  |  |  |
| Model1+"sarcopenia" | 0.907 (0.838–0.976) | 0.602(0.073–1.1323) | <0.001 | 0.044 (-0.027–0.114) | 0.114 |
| Non-dyslipidemia |  |  |  |  |  |
| Model1 | 0.602 (0.547–0.657) |  |  |  |  |
| Model1+"sarcopenia" | 0.636 (0.584–0.689) | 0.198 (0.006–0.389) | 0.157 | 0.006 (0.001–0.011) |  |
| Diabetes |  |  |  |  |  |
| Model1 | 0.832 (0.731–0.932) |  |  |  |  |
| Model1+"sarcopenia" | 0.839 (0.739–0.939) | 0.217(-0.374–0.809) | 0.119 | 0.018(-0.018–0.053) | 0.167 |
| Non-diabetes |  |  |  |  |  |
| Model1 | 0.611 (0.554–0.669) |  |  |  |  |
| Model1+"sarcopenia" | 0.646 (0.592–0.700) | 0.232 (0.042–0.422) | 0.096 | 0.008(0.002–0.015) | <0.001 |
| BMI≥24.0, kg/m^2^ |  |  |  |  |  |
| Model1 | 0.599 (0.519–0.679) |  |  |  |  |
| Model1+"sarcopenia" | 0.630 (0.555–0.705) | 0.153 (-0.119–0.426) | 0.271 | 0.003（-0.002–0.008） | 0.138 |
| BMI<24.0, kg/m^2^ |  |  |  |  |  |
| Model1 | 0.657 (0.59–0.724) |  |  |  |  |
| Model1+"sarcopenia" | 0.692 (0.623–0.76) | 0.310 (0.069–0.552) | 0.026 | 0.022（0.009–0.035） | <0.001 |
| eGFR 15**–<**60mL/  min/1.73 m² |  |  |  |  |  |
| Model1 | 0.793 (0.594–0.992) |  |  |  |  |
| Model1+"sarcopenia" | 0.793 (0.594–0.992) | 0.120(-0.655–0.895) | 0.391 | 0.006 (-0.003–0.015) | 0.112 |
| eGFR 60–89 mL/  min/1.73 m² |  |  |  |  |  |
| Model1 | 0.615 (0.559–0.671) |  |  |  |  |
| Model1+"sarcopenia" | 0.653 (0.602–0.704) | 0.255 (0.070–0.441) | 0.067 | 0.009 (0.003–0.016) | 0.003 |
| HUA |  |  |  |  |  |
| Model1 | 0.907 (0.844–0.971) |  |  |  |  |
| Model1+"sarcopenia" | 0.915 (0.855–0.975) | 0.280(-0.401–0.961) | 0.045 | 0.006 (-0.025–0.037) | 0.345 |
| Non-HUA |  |  |  |  |  |
| Model1 | 0.619 (0.563–0.675) |  |  |  |  |
| Model1+"sarcopenia" | 0.661 (0.609–0.713) | 0.276 (0.089–0.463) | 0.048 | 0.012 (0.004–0.019) | <0.001 |

Abbreviation: BMI, body mass index; CKD, chronic kidney disease; CI: confidence interval; HTN, hypertension; HR: hazard ratio; HUA, hyperuricemia.
